# Staffing, skill mix, shortage, and survival in the NICU and PICU: pediatrics quo vadis?

**DOI:** 10.1007/s00431-023-04970-w

**Published:** 2023-05-23

**Authors:** Sascha Meyer, Johannes Pelé Bay, Martin Poryo

**Affiliations:** 1 Franz-Lust Klinik für Kinder und Jugendliche, Städtisches Klinikum Karlsruhe, Moltkestraße 90, Gebäude 1, 76133 Karlsruhe, Germany; 2 Department of Pediatrics and Neonatology, Saarland University Medical Center, Homburg, Germany; 3 Department of Pediatric Cardiology, Saarland University Medical Center, Homburg, Germany

Dear Editor,

We read with great interest the report by Dr Genna et al. [1] exploring the association between nurse staffing levels or skill-mix and pediatric and neonatal critical deterioration events (e.g., pediatric intensive care unit (PICU)/neonatal intensive care unit (NICU) unplanned admissions, cardiac arrests, and failure-to-rescue, and mortality). Out of a total of 2319 studies, only 15 were included in the final analysis (7 PICU, 6 NICU, and 2 general pediatric wards). Most studies indicated that nursing skill-mix, increased working experience, or higher nursing degrees were associated with increased survival in PICU, while an association was seen between a shortage in nursing staffing levels and an increased mortality in the NICU and mechanically ventilated patients in the PICU.

With regard to this important issue, we would like to share our experience regarding physician staffing, level of expertise, and adequate/inadequate ventilation and neonatal outcome. In our audit, we included a cohort of very low birth weight (VLBW) infants who required intubation and invasive ventilation during the first 3 days of life [2]. During day shifts, one pediatric registrar receiving special neonatal training as well as three senior physicians covered the NICU. During night shifts (on both weekdays and weekends), only one pediatrics registrar was present in the NICU, but was backed-up by another senior physician/neonatologist on call. Allocation of nursing staff to the NICU was in accordance with all pertinent German regulatory specifications.

In line with previous studies [3], our audit showed that a relevant number of mechanically ventilated very premature infants experienced episodes of both hyperventilation and hypoventilation, and this pattern was significantly associated with a reduced presence of neonatologists. This finding — in line with results from other studies — stresses the importance of adequate staffing in NICUs, with regard to both nursing and physicians [1–5]. To improve this situation, both local (e.g., improved neonatal training) and political measures (e.g., adequate funding) will have to be implemented to provide a solid basis for the provision of safe and high-quality neonatal intensive care treatment. With regard to under-staffing in nursing and neonatal physician care, the employment of additional medical staff (e.g., physician assistant) may be helpful in improving the current shortage in staffing. Our findings are also congruent with studies of understaffing (nursing) in NICUs and the occurrence of other severe complications, most notably serious infections [1–5]. In addition, a non-significant association between neonatal morbidities and inadequate mechanical ventilation was noted, and a significant association of hypocapnic episodes and chronic lung disease was seen in our study population as well.

## References

[1] Genna C, Thekkan KR, Raymakers-Janssen P, Gawronski O (2023) Is nurse staffing associated with critical deterioration events on acute and critical care pediatric wards? A literature review. Eur J Pediatr. 10.1007/s00431-022-04803-2. Online ahead of print 10.1007/s00431-022-04803-2 36763191

[2] Röhr M, Poryo M, Bay J, Gortner L, Meyer S (2017). Episodes of hypo- and hypercapnia in a cohort of mechanically ventilated VLBW infants: the role of adequate staffing. Wien Med Wochenschr.

[3] van Kaam AH, De Jaegere AP, Rimensberger PC, Neovent Study Group (2013). Incidence of hypo- and hypercapnia in a cross-sectional European cohort of ventilated newborn infants. Arch Dis Child Fetal Neonatal Ed.

[4] Beltempo M, Patel S, Platt RW, Julien AS, Blais R, Bertelle V, Lapointe A, Lacroix G, Gravel S, Cabot M, Piedboeuf B (2023) Quebec investigators of the Canadian Neonatal Network; Quebec investigators of the Canadian Neonatal Network (CNN). Association of nurse staffing and unit occupancy with mortality and morbidity among very preterm infants: a multicentre study. Arch Dis Child Fetal Neonatal Ed fetalneonatal-2022–324414. 10.1136/archdischild-2022-324414. Online ahead of print 10.1136/archdischild-2022-324414 36609411

[5] Rogowski JA, Staiger D, Patrick T, Horbar J, Kenny M, Lake ET (2013). Nurse staffing and NICU infection rates. JAMA Pediatr.

